# Wrist and Thumb Kinematics During Sustained Smartphone Typing in Patients with Carpal Tunnel Syndrome: A Cross-Sectional Comparative Study

**DOI:** 10.3390/jcm15218604

**Published:** 2026-11-04

**Authors:** Kyoung-Sim Jung, Jin-Hwa Jung, Hwi-Young Cho, Tae-Sung In

**Affiliations:** 1 Department of Physical Therapy, Gimcheon University, Gimcheon 39528, Republic of Korea; 20240049@gimcheon.ac.kr; 2 Department of Occupational Therapy, Semyung University, Jecheon 27136, Republic of Korea; otsalt@semyung.ac.kr; 3 Department of Physical Therapy, College of Health Science, Gachon University, Incheon 21936, Republic of Korea

**Keywords:** carpal tunnel syndrome, smartphone, wrist, thumb, kinematics, angular velocity

## Abstract

**Objectives:** Carpal tunnel syndrome (CTS) is associated with impaired hand sensorimotor function, but wrist and thumb kinematics during sustained smartphone use remain unclear. This study compared joint posture, angular velocity, and time-related kinematic changes during a 30-min smartphone typing task between patients with CTS and healthy controls. **Methods:** Thirteen patients with CTS and 14 healthy controls completed a standardized 30-min two-handed smartphone typing task. Three-dimensional wrist and thumb kinematics were recorded, and mean joint angles and root mean square (RMS) angular velocities were calculated for wrist extension, wrist ulnar deviation, thumb metacarpophalangeal (MCP) flexion, MCP abduction, and interphalangeal (IP) flexion. Outcomes were analyzed over the entire task and across three 10-min intervals. **Results:** The CTS group showed greater wrist extension than the healthy control group (39.27 ± 9.31° vs. 27.99 ± 9.92°, *p* = 0.005) and less thumb MCP flexion (25.94 ± 8.43° vs. 32.66 ± 6.50°, *p* = 0.028). No significant between-group differences were found for the other joint angles or RMS angular velocities. No significant time effects or group × time interactions were observed. **Conclusions:** Patients with CTS demonstrated greater wrist extension and reduced thumb MCP flexion during sustained smartphone typing. These findings suggest that CTS-related kinematic differences may be more apparent in specific joint postures than in overall movement velocity.

## 1. Introduction

Carpal tunnel syndrome (CTS) is a common compressive neuropathy caused by compression of the median nerve at the wrist. It can result in pain, paresthesia, numbness, weakness of the hand, and impaired sensorimotor function. Consequently, patients with CTS may experience clumsiness during activities of daily living and unintentionally drop objects [1]. CTS has also been associated with reduced accuracy and stability of finger force control during sustained precision pinch tasks [2] and increased variability in finger movement and contact position during precision manipulation [3,4].

Handheld devices, including smartphones, are now widely used in daily life; however, the effects of prolonged device use on hand biomechanics remain incompletely understood [5]. A case–control study examining the association between smartphone use and CTS reported a significant association with smartphone use of ≥2 h/day, and use of ≥4 h/day remained significantly associated with CTS after adjustment for potential confounding variables [6]. These findings indicate an association between smartphone use and CTS but do not establish a direct causal relationship.

Mobile touchscreen devices differ from conventional electronic devices that use a keyboard and mouse because users must support the device while directly interacting with the screen. Consequently, the biomechanical demands on the upper extremity may vary according to device characteristics, usage conditions, and posture [7]. Previous research on the biomechanics of handheld device use has shown that the wrist frequently deviates from a neutral position during smartphone and tablet use and that the thumb carpometacarpal (CMC) and metacarpophalangeal (MCP) joints play important roles in device manipulation [5]. Thumb CMC and MCP joint motion and range of motion may also vary according to screen location and device size [5].

Wrist posture and repetitive finger movements may alter the mechanical environment within the carpal tunnel. Changes in wrist flexion–extension and radial–ulnar deviation affect carpal tunnel pressure, with greater wrist extension and radial deviation associated with increased pressure [8]. Typing itself has also been shown to increase carpal tunnel pressure compared with maintaining the same wrist posture without typing [8]. Repetitive keyboard use has been associated with acute morphological changes in the median nerve, including changes in cross-sectional area and swelling ratio, and maximum ulnar deviation has been associated with changes in median nerve cross-sectional area after 30 min of typing [9].

In addition to average joint posture, dynamic movement characteristics should be considered when evaluating biomechanical features associated with CTS. A cohort study using objective measurements of wrist movement demonstrated an exposure–response relationship between wrist angular velocity and the risk of CTS [10]. In a smartphone-based target manipulation task, patients with CTS also exhibited slower thumb movement than healthy participants and had particular difficulty with movements resembling thumb opposition [11]. These findings support the assessment of movement velocity together with average joint posture when investigating smartphone use in individuals with CTS.

Previous studies have examined the association between smartphone use and CTS [6], as well as wrist and thumb posture and movement during handheld device use [5,7]. Changes in carpal tunnel pressure with wrist posture [8], morphological changes in the median nerve after repetitive typing [9], and altered thumb movement during smartphone manipulation in patients with CTS [11] have also been reported. However, few studies have directly compared patients with CTS and healthy controls while both groups performed the same prolonged smartphone typing task and simultaneously assessed mean joint angles, angular velocity, and changes over time. In particular, limited information is available on wrist extension and ulnar deviation together with thumb MCP flexion, MCP abduction, and interphalangeal (IP) flexion during sustained smartphone use.

Therefore, this study aimed to compare the mean joint angles and root mean square (RMS) angular velocities of wrist extension, wrist ulnar deviation, thumb MCP flexion, thumb MCP abduction, and thumb IP flexion between patients with CTS and healthy controls during a 30-min smartphone typing task using a three-dimensional motion capture system.

## 2. Experimental Section

### 2.1. Study Design and Participants

This cross-sectional comparative study investigated three-dimensional wrist and thumb kinematics during sustained smartphone typing in patients with CTS and healthy controls. Participants were recruited between June and December 2022 from five orthopedic clinics in Gyeonggi Province, Republic of Korea. Recruitment posters were displayed at each clinic, and individuals who expressed an interest in participating were screened for eligibility according to the predefined inclusion and exclusion criteria. A total of 27 participants, including 13 patients with CTS and 14 healthy controls, were included in the study.

The inclusion criteria for the CTS group were as follows: (1) paresthesia, pain, and/or numbness in the median nerve distribution persisting for at least 3 months; (2) a positive finding on at least one provocative test, including Tinel’s sign, the Phalen test, or the median nerve compression test; (3) abnormal nerve conduction findings consistent with median neuropathy; and (4) a Boston Carpal Tunnel Questionnaire (BCTQ) Symptom Severity Scale (SSS) score > 1.5. Participants who met at least three of these four criteria were classified as having CTS [12]. For participants with bilateral CTS, the more symptomatic hand was selected for analysis [13]. For those with unilateral CTS, the affected hand was analyzed, whereas the dominant hand was analyzed in healthy controls. Participants were excluded if they had ulnar, radial, or proximal median neuropathy unrelated to CTS; a central nervous system disorder; a history of hand or wrist trauma or surgery; rheumatoid arthritis or osteoarthritis; diabetes mellitus; or a recent corticosteroid injection to the hand [14].

The sample size was estimated based on the study by Marquardt et al., which compared thumb joint kinematics between patients with CTS and healthy controls [12]. During thumb opposition, the MCP flexion–extension range of motion was 34° (95% confidence interval [CI], 22–46°) in the CTS group and 58° (95% CI, 47–68°) in the healthy control group. Standard deviations were estimated from the reported means and 95% CIs, yielding a Cohen’s d of approximately 1.43. Using this effect size, the required sample size was calculated using G*Power version 3.1.9.7 (Heinrich-Heine-Universität Düsseldorf, Düsseldorf, Germany), with a significance level of 0.05, statistical power of 0.80, and a two-tailed independent-samples *t*-test. The minimum required sample size was nine participants per group. The final analysis included 13 patients with CTS and 14 healthy controls. This study was approved by the Institutional Review Board of Gachon University (1044396-202110-HR-208-01). The trial was registered under registration number KCT0008310.

### 2.2. Experimental Procedures

All participants used the same smartphone, a Samsung Galaxy S21 (Samsung Electronics, Suwon, Republic of Korea). The device was equipped with a 6.2-inch Dynamic AMOLED 2X display (94.1 cm^2^; 1080 × 2400 pixels; aspect ratio, 20:9), measured 151.7 × 71.2 × 7.9 mm, and weighed 169 g.

Participants sat on a height-adjustable chair with their knees flexed to approximately 90° and both feet flat on the floor. The shoulders were kept relaxed, and the table height was adjusted according to each participant’s body dimensions so that the elbows were flexed to approximately 90° and the forearms could rest comfortably on the table.

After placement of the reflective markers and completion of static calibration, all participants were provided with the same printed text passage. They were instructed to type the text on the smartphone using both hands for 30 min. No restrictions were imposed on which fingers were used for typing. If a participant completed the entire text before the end of the 30-min period, the participant restarted from the beginning and continued typing until the full 30 min had elapsed. Typing speed was not measured or controlled, and the number of characters typed and the specific fingers used for typing were not recorded. Nail length was not quantitatively measured; however, none of the participants had polished nails or excessively long nails that could substantially interfere with smartphone typing.

Wrist and thumb kinematic data were recorded continuously throughout the 30-min task. Mean joint angles and RMS angular velocities were calculated over the entire 30-min period. To assess changes over time, the data were further divided into three intervals: 0–10, 10–20, and 20–30 min. Values for each interval represented the mean joint angle and RMS angular velocity calculated from all data collected during that 10-min interval rather than measurements obtained at a single time point.

### 2.3. Three-Dimensional Kinematic Measurements

Three-dimensional wrist and thumb kinematics were recorded using an optical motion capture system consisting of 10 infrared cameras (Raptor-E, Motion Analysis Inc., Santa Rosa, CA, USA). Raw three-dimensional coordinate data were collected at a sampling frequency of 100 Hz and processed using ORTHOTRAK software (version 6.2.4, Motion Analysis Inc., Santa Rosa, CA, USA).

A total of 13 physical retroreflective markers were attached to anatomical landmarks on the tested forearm, wrist, dorsum of the hand, and thumb. The forearm marker was placed on the dorsal aspect of the mid-forearm approximately 8–10 cm proximal to the wrist. To establish anatomical references for the wrist, markers were placed over the radial and ulnar styloid processes, with an additional marker positioned over the capitate at the dorsal center of the wrist between the two styloid processes.

Three markers were placed on the dorsum of the hand to define the hand segment. These markers were positioned near the head of the second metacarpal on the index-finger side, over the central dorsum of the hand between the third and fourth metacarpals, and near the head of the fifth metacarpal on the little-finger side.

Six markers were used to measure thumb kinematics. The first marker was positioned at the base of the first metacarpal near the CMC joint, and the second was placed on the proximal phalanx immediately distal to the MCP joint. The third marker was placed at the distal end of the proximal phalanx immediately proximal to the IP joint, and the fourth was positioned on the distal phalanx immediately distal to the IP joint. The fifth marker was placed between the middle and distal portions of the distal phalanx, and the sixth was positioned near the base of the thumbnail toward the thumb tip.

During static calibration, two virtual markers were generated in ORTHOTRAK to define thumb MCP and IP joint motion. The first virtual marker was used to define the flexion–extension and abduction–adduction axes of the thumb MCP joint, whereas the second was used to define the flexion–extension axis of the thumb IP joint.

Wrist extension and ulnar deviation angles were calculated from the relative motion between the forearm and hand segments. For ease of interpretation, the signs of the original wrist flexion–extension and radial–ulnar deviation angles were reversed so that positive values represented wrist extension and ulnar deviation, respectively; negative values therefore represented wrist flexion and radial deviation. Thumb MCP flexion and abduction angles were calculated from the relative motion between the first metacarpal and proximal phalanx, whereas thumb IP flexion was calculated from the relative motion between the proximal and distal phalanges.

The kinematic variables analyzed in this study were wrist extension, wrist ulnar deviation, thumb MCP flexion, thumb MCP abduction, and thumb IP flexion.

### 2.4. Data Processing

Raw coordinate data collected at 100 Hz were low-pass-filtered using a Butterworth filter with a cutoff frequency of 10 Hz before joint angles were calculated.

For each participant, the mean joint angles for wrist extension, wrist ulnar deviation, thumb MCP flexion, thumb MCP abduction, and thumb IP flexion were calculated over the entire 30-min task. The data were also divided into 0–10, 10–20, and 20–30 min intervals, and the mean joint angle was calculated separately for each interval.

Joint angular velocity was derived from the change in joint angle over time. To quantify the magnitude of movement velocity irrespective of movement direction, the RMS of the angular velocity was calculated for each joint and expressed in °/s. RMS angular velocity was calculated over the entire 30-min task and separately for the 0–10, 10–20, and 20–30 min intervals for each participant.

### 2.5. Data Analysis

All statistical analyses were performed using IBM SPSS Statistics version 23.0 (IBM Corp., Armonk, NY, USA). Continuous variables are presented as mean ± standard deviation. Age and BMI were compared between groups using independent-samples *t*-tests, while sex and tested-hand distributions were compared using Pearson’s chi-square test and Fisher’s exact test, respectively. Between-group differences in joint angles and RMS angular velocities calculated over the entire 30-min task were analyzed using independent-samples *t*-tests. Separately, time-related changes based on the three 10-min intervals were assessed using a 2 × 3 mixed-design repeated-measures ANOVA, with group (CTS vs. healthy control) as the between-subject factor and time interval (0–10, 10–20, and 20–30 min) as the within-subject factor. Significant time effects or group × time interactions were followed by Bonferroni-adjusted comparisons, as appropriate. Normality and homogeneity of variance were assessed using the Shapiro–Wilk test and Levene’s test, respectively, and the assumption of sphericity for the repeated-measures ANOVA was assessed using Mauchly’s test. All relevant assumptions were satisfied. All tests were two-tailed, with statistical significance set at *p* < 0.05.

## 3. Results

### 3.1. General Characteristics of Participants

A total of 27 participants were included in the study, comprising 13 patients with CTS and 14 healthy controls. The mean age was 32.1 ± 7.6 years in the CTS group and 35.4 ± 9.5 years in the healthy control group, with no significant between-group difference (*p* = 0.334). BMI was also comparable between groups (23.5 ± 5.8 kg/m^2^ vs. 21.7 ± 5.0 kg/m^2^, respectively; *p* = 0.392). The CTS group included seven women and six men, whereas the healthy control group included eight women and six men; sex distribution did not differ significantly between groups (*p* = 0.863). The distribution of the tested hand was also comparable, with right/left hand distributions of 9/4 in the CTS group and 11/3 in the healthy control group (*p* = 0.678).

In the CTS group, the mean symptom duration was 1.6 ± 0.8 years. Mean BCTQ SSS and Functional Status Scale scores were 2.2 ± 0.5 and 1.8 ± 0.8, respectively, and the mean pain visual analogue scale score was 3.3 ± 3.5. Three participants with CTS (23.1%) had bilateral CTS (Table 1). Of the 13 participants with CTS, nine fulfilled all four diagnostic criteria, whereas four fulfilled three of the four criteria. All 13 participants fulfilled the symptom-duration and provocative-test criteria, 10 had abnormal nerve conduction findings, and 12 had a BCTQ SSS score > 1.5. Among the four participants who fulfilled three criteria, three did not meet the nerve conduction criterion and one did not meet the BCTQ SSS criterion. Among the 10 participants with abnormal nerve conduction findings, electrodiagnostic severity was classified as mild in four, moderate in five, and severe in one.

### 3.2. Between-Group Differences in Joint Angles and RMS Angular Velocities During the 30-min Smartphone Task

During the 30-min smartphone typing task, mean wrist extension was significantly greater in the CTS group than in the healthy control group (39.27 ± 9.31° vs. 27.99 ± 9.92°, respectively; t = 3.040, *p* = 0.005). In contrast, mean thumb MCP flexion was significantly lower in the CTS group than in the healthy control group (25.94 ± 8.43° vs. 32.66 ± 6.50°, respectively; t = −2.332, *p* = 0.028). No significant between-group differences were observed in wrist ulnar deviation, thumb MCP abduction, or thumb IP flexion (all *p* > 0.05). No significant between-group differences were observed in the 30-min RMS angular velocities of wrist extension, wrist ulnar deviation, thumb MCP flexion, thumb MCP abduction, or thumb IP flexion (all *p* > 0.05) (Table 2). Negative mean wrist ulnar deviation values, indicating that the average wrist position was in radial deviation, were observed in 6 of 13 participants (46.2%) in the CTS group and 8 of 14 participants (57.1%) in the healthy control group.

### 3.3. Changes in Joint Angles and RMS Angular Velocities Across Smartphone-Use Intervals

Analysis of mean joint angles across the 0–10, 10–20, and 20–30 min intervals revealed a significant main effect of group for wrist extension (F = 9.24, *p* = 0.005), with the CTS group exhibiting greater wrist extension overall than the healthy control group. A significant group main effect was also observed for thumb MCP flexion (F = 5.44, *p* = 0.028), with the CTS group exhibiting less flexion overall. However, neither the main effect of time nor the group × time interaction was significant for any joint, including wrist extension and thumb MCP flexion (all *p* > 0.05). No significant group main effects were observed for wrist ulnar deviation, thumb MCP abduction, or thumb IP flexion (all *p* > 0.05). For RMS angular velocity, no significant group main effects, time main effects, or group × time interactions were observed for wrist extension, wrist ulnar deviation, thumb MCP flexion, thumb MCP abduction, or thumb IP flexion (all *p* > 0.05) (Table 3).

## 4. Discussion

This study compared wrist and thumb kinematics between patients with CTS and healthy controls during the same 30-min smartphone typing task. The principal findings were that patients with CTS exhibited greater wrist extension and less thumb MCP flexion than healthy controls. In contrast, no significant between-group differences were observed in wrist ulnar deviation, thumb MCP abduction, or thumb IP flexion, and RMS angular velocity did not differ between groups for any joint. In addition, neither mean joint angles nor RMS angular velocities showed significant time effects or group × time interactions over the 30-min task.

### 4.1. Posture-Specific Rather than Velocity-Related Kinematic Differences in CTS

Mean wrist extension was significantly greater in the CTS group than in the healthy control group (39.27° vs. 27.99°, respectively). Handheld device use can place the wrist away from a neutral posture, often involving wrist extension and ulnar deviation, and wrist posture varies according to device size, orientation, and method of use [5]. Studies examining wrist posture and carpal tunnel pressure during typing have shown that greater wrist extension is associated with increased carpal tunnel pressure [8]. Thus, the greater wrist extension observed in the CTS group may represent a posture associated with greater mechanical loading of the carpal tunnel during smartphone typing.

Although wrist ulnar deviation did not differ significantly between groups, the mean value was numerically greater in the CTS group than in the healthy control group (3.67° vs. 1.40°). In healthy adults performing prolonged keyboard typing, greater maximum ulnar deviation was associated with a larger change in median nerve cross-sectional area after 30 min [9]. In addition, a study simulating smartphone-related hand movements found that the combination of thumb opposition and wrist ulnar deviation produced the greatest median nerve deformation and translational movement [15]. Although mean ulnar deviation was numerically greater in the CTS group, participants in both groups showed considerable variability in wrist deviation direction, with mean positions ranging from radial to ulnar deviation. The between-group difference was not statistically significant. Thus, the difference in wrist posture between the CTS and healthy control groups was more evident in wrist extension than in ulnar deviation.

For the thumb, mean MCP flexion was significantly lower in the CTS group than in the healthy control group (25.94° vs. 32.66°, respectively). Previous studies of thumb kinematics in CTS have demonstrated reduced thumb opposition and circumduction [12]. Similarly, in a smartphone task requiring thumb movements in multiple directions, patients with CTS showed poorer performance particularly for movements resembling thumb opposition [11]. Taken together, the reduced MCP flexion observed in the present study may reflect an altered thumb movement strategy used by patients with CTS during smartphone typing. No significant between-group differences were observed in thumb MCP abduction or IP flexion. MCP abduction was numerically lower in the CTS group (*p* = 0.057). Thumb joint posture during smartphone use can vary according to screen location, movement direction, device size, and grip configuration [5,16,17,18]. Furthermore, handheld device manipulation appears to rely more heavily on the CMC and MCP joints, with a relatively smaller contribution from the IP joint [5]. To reduce variability attributable to these external factors, all participants in the present study used the same smartphone and a two-handed grip. Under these standardized conditions, the clearest between-group difference in thumb posture was observed in MCP flexion.

No significant between-group differences were found in RMS angular velocity for any of the joints examined. A previous smartphone-based study reported lower mean and maximum thumb movement velocities in patients with CTS than in healthy controls [11]. That study, however, used a goal-directed task in which participants moved the thumb toward specified targets on the screen, whereas the present study quantified joint-specific RMS angular velocity during continuous typing over 30 min. Thus, the reduction in movement speed previously observed in CTS may be less apparent during repetitive, continuous smartphone typing. Long-term occupational exposure to higher wrist angular velocities has also been associated with an increased risk of developing CTS [10]. This finding indicates that repeated exposure to high angular velocity may represent a biomechanical risk factor for CTS; it does not imply that individuals who already have CTS necessarily move their wrists faster than healthy individuals during the same task. In the present study, both groups performed repetitive typing using the same smartphone and two-handed configuration. In addition, RMS angular velocity summarizes movement velocity over the entire task and may therefore be less sensitive to brief or direction-specific differences in movement speed. These task and analytical characteristics may partly explain the absence of significant between-group differences in RMS angular velocity.

Overall, the kinematic differences between the CTS and healthy control groups during smartphone typing were more apparent in specific joint postures, particularly wrist extension and thumb MCP flexion, than in overall joint movement velocity. From a clinical perspective, the greater wrist extension and reduced thumb MCP flexion observed in the CTS group may provide useful kinematic features to consider when evaluating smartphone-use posture in individuals with CTS. In particular, greater wrist extension may warrant attention because wrist extension has been associated with increased carpal tunnel pressure. These findings may support ergonomic assessment and education aimed at reducing excessive wrist extension during smartphone use. However, the present study did not evaluate whether modifying these postures reduces CTS symptoms, and further interventional studies are needed to determine their clinical effectiveness.

### 4.2. Temporal Stability of Wrist and Thumb Kinematics During Sustained Smartphone Typing

When the 30-min smartphone typing task was divided into 0–10, 10–20, and 20–30 min intervals, significant group main effects were observed for wrist extension and thumb MCP flexion, whereas no significant time effects or group × time interactions were identified for any joint. These findings indicate that the between-group differences in wrist extension and thumb MCP flexion did not emerge during a particular interval or progressively increase over time, but remained relatively consistent throughout the 30-min task. A previous study examining prolonged keyboard typing reported increases in median nerve cross-sectional area and swelling ratio after 30 and 60 min, followed by a return toward baseline after 30 min of rest [9]. These findings suggest that time-dependent changes in internal tissues such as the median nerve may occur during repetitive input tasks even in the absence of marked changes in externally measured joint kinematics. Accordingly, the absence of a significant time effect for joint angles in the present study should be interpreted as indicating that no clear temporal change in joint posture occurred during the 30-min task. Similarly, no significant time effects or group × time interactions were observed for RMS angular velocity. Thus, joint movement velocity did not show a systematic increase or decrease over the 30-min typing task in either group. Taken together, the present findings indicate that progressive kinematic changes were not evident during 30 min of smartphone typing, while the principal between-group differences remained those in wrist extension and thumb MCP flexion.

### 4.3. Limitations

This study has several limitations. First, the relatively small sample of 13 patients with CTS and 14 healthy controls limits the generalizability of the findings to the broader CTS population. In addition, the inclusion of participants who fulfilled three of the four diagnostic criteria may have introduced some diagnostic heterogeneity within the CTS group. Second, because of the cross-sectional design, it cannot be determined whether the greater wrist extension and reduced thumb MCP flexion observed in the CTS group preceded CTS or represented compensatory strategies that developed after symptom onset. Third, the experimental setting was standardized using the same smartphone, seated posture, and two-handed configuration for all participants. In everyday smartphone use, however, device size, grip configuration, and body posture vary considerably. Therefore, the present findings should be generalized with caution to broader CTS populations and real-world smartphone-use conditions that differ from the standardized experimental setting used in this study. In addition, the specific fingers used for typing were not restricted, and typing speed, the number of characters typed, and the specific fingers used were not recorded. These factors may have influenced typing performance and joint kinematics and therefore represent potential confounders. Future studies should measure and more carefully control these typing-related factors. Nail length was not quantitatively measured; therefore, subtle biomechanical effects associated with individual differences in nail length cannot be completely excluded. Thumb CMC joint kinematics were not directly assessed in this study. Given the important role of the CMC joint in thumb opposition and handheld device manipulation, the absence of CMC joint measurements limits the interpretation of whether the observed differences in thumb MCP flexion reflect isolated MCP joint alterations or broader changes in thumb movement strategies involving the CMC joint. Future studies should incorporate three-dimensional CMC joint kinematics to provide a more comprehensive assessment of thumb movement in individuals with CTS. Fourth, only joint kinematics were measured. Carpal tunnel pressure, median nerve cross-sectional area or deformation, and muscle activity were not assessed concurrently. Consequently, the direct relationship between the wrist and thumb postures observed in the CTS group and the actual mechanical loading of the median nerve could not be determined. Future studies with larger samples should combine joint kinematic measurements with median nerve ultrasonography and muscle activity assessments.

## 5. Conclusions

During a 30-min smartphone typing task, patients with CTS exhibited significantly greater wrist extension and less thumb MCP flexion than healthy controls. No significant between-group differences were observed in wrist ulnar deviation, thumb MCP abduction, thumb IP flexion, or RMS angular velocity for any joint. In addition, none of the kinematic variables showed significant time-related changes during the 30-min task.

These findings suggest that kinematic differences during smartphone use in patients with CTS may be more evident in specific joint postures, particularly wrist extension and thumb MCP flexion, than in overall movement velocity. Future studies integrating joint kinematics with structural and physiological assessments of the median nerve are warranted to clarify the clinical significance of these postural differences.

## Figures and Tables

**Table 1:** General characteristics of the participants.

Variable	CTS Group (*n* = 13)	Healthy Control Group (*n* = 14)	*p*-Value
Age, years	32.1 ± 7.6	35.4 ± 9.5	0.334
BMI, kg/m^2^	23.5 ± 5.8	21.7 ± 5.0	0.392
Sex, female/male	7/6	8/6	0.863
Symptom duration, years	1.6 ± 0.8	–	–
BCTQ Symptom Severity Scale	2.2 ± 0.5	–	–
BCTQ Functional Status Scale	1.8 ± 0.8	–	–
Pain VAS, 0–10	3.3 ± 3.5	–	–
Measured hand, R/L	9/4	11/3	0.678
Bilateral CTS, n (%)	3 (23.08)	–	–

Values are presented as mean ± standard deviation or number (%). BMI, body mass index; BCTQ, Boston Carpal Tunnel Questionnaire; VAS, visual analogue scale; R, right; L, left. Age and BMI were compared using independent-samples *t*-tests; sex was compared using the Pearson chi-square test; measured hand was compared using Fisher’s exact test.

**Table 2:** Between-Group Comparison of Joint Angles and RMS Angular Velocities during the 30-min Smartphone Typing Task.

Outcome	Variable	CTS Group (*n* = 13)	Healthy Control Group (*n* = 14)	t	*p*	Effect Size Cohen’s d (95%CI)
Mean joint angle (°)	Wrist extension	39.27 ± 9.31	27.99 ± 9.92	3.040	**0.005**	1.17 (0.35, 1.99)
	Wrist ulnar deviation	3.67 ± 9.48	1.40 ± 8.41	0.657	0.517	0.25 (−0.50, 1.01)
	Thumb MCP flexion	25.94 ± 8.43	32.66 ± 6.50	−2.332	**0.028**	−0.90 (−1.69, −0.10)
	Thumb MCP abduction	21.39 ± 7.79	25.94 ± 3.39	−1.994	0.057	−0.77 (−1.55, 0.02)
	Thumb IP flexion	34.43 ± 9.74	30.27 ± 9.78	1.107	0.279	0.43 (−0.34, 1.19)
RMS angular velocity (°/s)	Wrist extension	14.59 ± 6.66	14.00 ± 4.30	0.274	0.787	0.11 (−0.65, 0.86)
	Wrist ulnar deviation	12.38 ± 5.70	9.69 ± 3.28	1.516	0.142	0.58 (−0.19, 1.36)
	Thumb MCP flexion	39.28 ± 15.06	46.04 ± 13.77	−1.219	0.234	−0.47 (−1.24, 0.30)
	Thumb MCP abduction	37.26 ± 10.56	36.57 ± 8.40	0.190	0.851	0.07 (−0.68, 0.83)
	Thumb IP flexion	42.44 ± 13.25	38.03 ± 7.73	1.065	0.297	0.41 (−0.35, 1.17)

Values are presented as mean ± standard deviation. MCP, metacarpophalangeal; IP, interphalangeal; RMS, root mean square. Bold *p*-values indicate statistical significance (*p* < 0.05).

**Table 3:** Repeated-Measures ANOVA of Joint Angles and RMS Angular Velocities across Smartphone Typing Intervals.

Outcome Measure	Variable	Group Effect, F (*p*)	Time Effect, F (*p*)	Group × Time Interaction, F (*p*)
Mean joint angle (°)	Wrist extension	**9.24 (0.005)**	0.37 (0.690)	0.43 (0.655)
	Wrist ulnar deviation	0.43 (0.517)	0.46 (0.635)	0.23 (0.798)
	Thumb MCP flexion	**5.44 (0.028)**	0.02 (0.983)	0.10 (0.907)
	Thumb MCP abduction	3.98 (0.057)	2.84 (0.068)	0.04 (0.961)
	Thumb IP flexion	1.23 (0.279)	0.33 (0.723)	0.00 (0.995)
RMS angular velocity (°/s)	Wrist extension	0.075 (0.787)	1.260 (0.292)	0.092 (0.912)
	Wrist ulnar deviation	2.297 (0.142)	0.772 (0.467)	0.294 (0.746)
	Thumb MCP flexion	1.527 (0.228)	1.472 (0.239)	0.711 (0.496)
	Thumb MCP abduction	0.036 (0.851)	0.533 (0.590)	1.402 (0.256)
	Thumb IP flexion	1.135 (0.297)	1.181 (0.315)	1.897 (0.161)

ANOVA results are presented as F (*p*). MCP, metacarpophalangeal; IP, interphalangeal; RMS, root mean square. Bold values indicate statistical significance (*p* < 0.05).

## Data Availability

The data presented in this study are available from the corresponding author upon reasonable request. The data are not publicly available due to privacy and ethical restrictions.
